# One-year and three-year mortality prediction in adult major blunt trauma survivors: a National Retrospective Cohort Analysis

**DOI:** 10.1186/s13049-018-0497-y

**Published:** 2018-04-18

**Authors:** Ting Hway Wong, Nivedita Vikas Nadkarni, Hai V. Nguyen, Gek Hsiang Lim, David Bruce Matchar, Dennis Chuen Chai Seow, Nicolas K. K. King, Marcus Eng Hock Ong

**Affiliations:** 1 Department of General Surgery, Singapore General Hospital / Duke-National University of Singapore Medical School, Outram Road, Singapore, 169608 Republic of Singapore; 2 Duke-National University of Singapore Medical School, Singapore, Singapore; 3 School of Pharmacy, Memorial University of Newfoundland, St. John’s, Canada; 4 National Registry of Diseases Office, Health Promotion Board, Singapore, Singapore; 5 Department of Geriatric Medicine, Singapore General Hospital / Duke-National University of Singapore Medical School, Singapore, Singapore; 6 Department of Neurosurgery, National Neuroscience Institute, Singapore, Singapore; 7 Department of Emergency Medicine, Singapore General Hospital / Duke-National University of Singapore Medical School, Singapore, Singapore

**Keywords:** Blunt trauma injury long-term survival adult

## Abstract

**Background:**

Survivors of trauma are at increased risk of dying after discharge. Studies have found that age, head injury, injury severity, falls and co-morbidities predict long-term mortality. The objective of our study was to build a nomogram predictor of 1-year and 3-year mortality for major blunt trauma adult survivors of the index hospitalization.

**Methods:**

Using data from the Singapore National Trauma Registry, 2011–2013, we analyzed adults aged 18 and over, admitted after blunt injury, with an injury severity score (ISS) of 12 or more, who survived the index hospitalization, linked to death registry data. The study population was randomly divided 60/40 into separate construction and validation datasets, with the model built in the construction dataset, then tested in the validation dataset. Multivariable logistic regression was used to analyze 1-year and 3-year mortality.

**Results:**

Of the 3414 blunt trauma survivors, 247 (7.2%) died within 1 year, and 551 (16.1%) died within 3 years of injury. Age (OR 1.06, 95% CI 1.05–1.07, *p* < 0.001), male gender (OR 1.53, 95% CI 1.12–2.10, *p* < 0.01), low fall from 0.5 m or less (OR 3.48, 95% CI 2.06–5.87, p < 0.001), Charlson comorbidity index of 2 or more (OR 2.26, 95% CI 1.38–3.70, p < 0.01), diabetes (OR 1.31, 95% CI 1.68–2.52, *p* = 0.04), cancer (OR 1.76, 95% CI 0.94–3.32, *p* = 0.08), head and neck AIS 3 or more (OR 1.79, 95% CI 1.13–2.84, *p* = 0.01), length of hospitalization of 30 days or more (OR 1.99, 95% CI 1.02–3.86, *p* = 0.04) were predictors of 1-year mortality. This model had a c-statistic of 0.85. Similar factors were found significant for the model predictor of 3-year mortality, which had a c-statistic of 0.83. Both models were validated on the second dataset, with an overall accuracy of 0.94 and 0.84 for 1-year and 3-year mortality respectively.

**Conclusions:**

Adult survivors of major blunt trauma can be risk-stratified at discharge for long-term support.

## Background

Long-term mortality for survivors of trauma is an important indicator of the societal impact of trauma, as survivors of the index hospitalization have an increased risk of dying in the post-discharge period [1–6]. Healthcare resource utilization is higher for survivors of trauma after discharge [7], and the quality of care received during the index hospitalization can improve long-term survival [8]. Age, head injury, injury severity, low falls, co-morbidities and discharge destination are associated with long-term mortality [6, 9, 10].

Our hypothesis was that, for adult blunt trauma patients sustaining major injury, and who survived the index hospitalization, different risk factors would contribute differentially to 1-year and 3-year mortality, and that patients with multiple risk factors would be at higher risk than patients with single risk factors. Hence, the goal of our study was to construct predictive nomograms to predict 1-year and 3-year mortality for adult survivors of major blunt trauma, defined in a recent study as injury severity score of 12 or more [11], with the eventual objective of targeting high-risk survivors for intervention. We examined the location and certified causes of death for these patients.

## Methods

Singapore is an Asian urban country with a long life expectancy, a centralized national pre-hospital ambulance system [12], and a mixed public healthcare system [13]. A retrospective cohort study was performed using data from the Singapore National Trauma Registry [10, 14] from January 2011 to December 2013, of patients aged 18 years or older, admitted to a public hospital via the emergency department, with an injury severity score of 12 or more after sustaining blunt trauma [11], and who survived the index hospitalization.

The registry inclusion criteria, data collection, data cleaning and data quality audit processes have been described in previous studies [10, 14]. The data was matched to death information from the registry of births and deaths, provided by the National Registry of Diseases Office, till December 2016, to obtain long-term survival, location and causes of death. Patients with isolated burns, drowning, and hanging, were excluded. Non-residents were excluded from our study, as the death registry captures vital information for residents only.

Our primary outcome of interest was 1-year mortality, and the secondary outcome was 3-year mortality. To compare the outcomes from our study population with the general Singapore resident population, we calculated the expected age-sex standardized 1- and 3-year mortality for the study population and for high-risk sub-groups, by applying the study population age- and sex- distribution from the general Singapore resident population, as obtained from the Singapore Department of Statistics. Death registry data was used to extract location and causes of death, as documented in the death certificate.

The following variables were extracted from the National Trauma Registry: age, race, gender, mechanism of injury (low falls, using standardised conversion guidelines from patient histories, defined as 0.5 m or less based on prior research [10]; non-low falls, motor vehicle injuries, other blunt injury), co-morbidities (Charlson co-morbidity index, individual co-morbidities affecting 3% or more of our study population), injury severity scores (ISS, new injury severity score / NISS, Revised Trauma Score / RTS, abbreviated injury scale / AIS of 3 or more for each ISS region, anatomical polytrauma – AIS score of 3 or more in at least two ISS regions), length of stay (intensive care unit, high-dependency unit, overall hospitalization); and treatment factors (blood transfusions – indicator of haemorrhagic shock and / or coagulopathy; operations grouped by surgical table, and complications).

Age was analysed as a continuous variable, after confirming linear association with log odds. Sensitivity analysis was performed using age in bands, to include age cut-offs commonly cited in the literature [15–18].

The Charlson Comorbidity Index was the primary measure of comorbidities, calculated based on the ICD-9 codes in the registry using STATA 13. In addition to the overall Charlson index for each patient, individual pre-existing / co-morbid conditions present in 3% or more of our study population were included as separate variables in the analysis.

Surgical procedures, coded by Ministry of Health table of surgical procedure [13], were grouped by surgical region, and the following groups that had been performed in 3% or more of our study population were included in our analysis: neurosurgery (including insertion of intracranial pressure monitors), orthopaedic, laparotomy and tracheostomy.

Complications present in 3% or more of our study population were included in the analysis. The ICD-9 codes were classified by disease group using the Clinical Classifications Software tool by the Agency for Healthcare Research and Quality [19].

The study population was randomly divided 60/40 into separate construction and validation datasets, with the model built in the construction dataset, then tested in the validation dataset.

Predictors that were significant in the univariate regression at 5% level of significance were entered into the multivariable regression. Sensitivity analyses including variables that were considered to be of potential clinical significance (race, discharge destination, injury severity scores, pneumonia, urinary tract infection) were performed. Additional sensitivity analyses were performed to exclude patients that were transferred in, or whose admission was delayed for more than 24 h. Logistic regression was used to analyse 1-year and 3-year mortality, with stepwise method for variable selection. Akaike Information Criterion was used to choose the final models. The Hosmer-Lemeshow goodness-of-fit test was performed to check model adequacy. Analysis was performed using STATA version 13 and R version 3.2.2. The rms package in R was used to generate the scores in the nomogram, based on the beta-estimates from the logistic regression, to obtain the “points” per predictive factor. The sum of these scores (“total points”) correspond to the predicted probabilities of death on the corresponding nomogram [20].

There were nine patients with missing data for clinical variables (Glasgow coma scale and / or respiratory rate); these patients were included in the final analysis as the two missing clinical variables were not used in the final model. For the 50 patients with unknown fall heights (2.6% of all falls), these falls were categorised as non-low falls.

At the time of study, the national ambulance service (the Singapore Civil Defence Force ambulance) sent patients to the nearest public hospital. Hence, patients conveyed by other modes of transport (private ambulance or private vehicle) to a private hospital would have been excluded from this study. However, this would lead to only a limited bias on the capture of minor injuries as public ambulance usage is high [12].

## Results

Four thousand four hundred thirty-seven blunt trauma survivors had an injury severity score of 12 or more during the study period, and 1023 non-residents were excluded. Of the 3414 Singapore resident blunt trauma survivors, 247 (7.2%) died within a year of injury, and 551 (16.1%) died within 36 months of injury. Majority were male (2299, 67.3%), the commonest mechanism of injury was a low fall (1556, 45.6%), and the mean age was 59.8 years of age (Table 1). Most were discharged home (2406, 70.5%).

**Table 1:** Characteristics of Adult Blunt Trauma Survivors (*n* = 3414)

		Number (%) / mean (SD) / median (IQR)
Demographics	Male	2299 (67.3)
	Age, mean (SD)	59.8 (21.3)
Injury Severity	ISS, median (IQR)	17 (14–25)
	NISS, median (IQR)	22 (17–29)
	Head and Neck AIS ≥3	2137 (62.3)
	Face AIS ≥3	36 (1.1)
	Thorax AIS ≥3	942 (27.6)
	Abdomen AIS ≥3	309 (9.1)
	Extremity AIS ≥3	601 (17.6)
	Polytrauma (anatomical: AIS of ≥ 3 in ≥ 2 body regions)	585 (17.1)
	RTS, median (IQR)	7.8 (7.8–7.8)
	GCS, median (IQR)	15 (14–15)
Mechanism of Injury	Motor Vehicle	208 (6.1)
	Motorcycle	723 (21.2)
	Pedestrian	124 (3.6)
	Other Road Injury	211 (6.2)
	Low fall (0–0.5 m, inclusive)	1556 (45.6)
	Non-low-fall (more than 0.5 m or unknown)	394 (11.5)
	Assault	74 (2.2)
	Blunt-other	124 (3.6)
Co-morbidities	Charlson Comorbidity Index (CCI) score 0	2434 (71.9)
	CCI 1	649 (19.2)
	CCI 2	206 (6.1)
	CCI 3 and above	98 (2.9)
	Diabetes mellitus	547 (16.0)
	Hyperlipidemia	404 (11.8)
	Hypertension	246 (7.2)
	Cancer	107 (3.1)
	Alcohol use prior to injury	251 (7.4)
Blood Products	Number of patients requiring blood transfusion	261 (7.6)
	Number of units of blood transfused, mean (SD)	0.3 (5.2)
	Number of patients requiring other blood products	37 (1.1)
Surgical Procedures	Neurosurgical	487 (14.3)
	Orthopedic	899 (26.3)
	Laparotomy	105 (3.1)
	Tracheostomy	100 (2.9)
Complications	Pneumonia	94 (2.8)
	Fluid and electrolyte disorders	133 (3.9)
	Urinary Tract Infection	145 (4.2)
Admission	Number of patients admitted to the intensive care unit at any time during admission	753 (22.1)
	Number of patients admitted to high dependency (including intermediate care) at any time during admission	1429 (41.9)
	Total length of stay in hospital, median number of days (IQR)	9.4 (4.2–21.3)
	Transferred in from another hospital^a^	131 (3.8)
	Admitted > 24 h post-injury^a^	167 (4.9)
Mortality	1-year mortality	247 (7.2)
	3-year mortality	551 (16.1)
Discharge Destination	Home	2406 (70.5)
	Rehabilitation	614 (18.0)
	Transferred to other facility, institution or step-down care	394 (11.5)

^a^includes 8 patients who were transferred from another hospital and admitted > 24 h post-injury

The demographic and clinical characteristics of the subjects in the derivation and validation samples were similar on all characteristics, except there was an insignificant difference in proportion of patients with a face AIS score of 3 or more, likely due to the overall low incidence of this type of injury.

### Predictors of 1-year and 3-year mortality

Age, ISS, NISS, head and neck AIS of 3 or more, low fall, co-morbidities (Charlson index of 2, Charlson index of 3 or more, diabetes mellitus, cancer), fluid and electrolyte disturbances, and total hospitalization period of 30 days or more, were significant univariate predictors of 1-year mortality. The following factors reduced the likelihood of 1-year mortality in univariate analysis: male gender, polytrauma (anatomical), AIS of 3 or more for the thorax, abdomen and extremity regions, all high-velocity mechanisms of injury (non-low fall, motor vehicle, motorcycle), undergoing an orthopedic operation, and receiving a blood transfusion (Table 2). Discharge destination, race, revised trauma score, intensive care unit length of stay, alcohol ingestion prior to injury, in-hospital complications – urinary tract infection and pneumonia, were not significant predictors of 1-year mortality.

**Table 2:** Predictors of 1-year and 3-year Mortality

	1-year Mortality						3-year Mortality					
	Univariate			Multivariable			Univariate			Multivariable		
	Odds Ratio	[95% Conf. Interval]	*P*-value	Odds Ratio	[95% Conf. Interval]	P-value	Odds Ratio	[95% Conf. Interval]	P-value	Odds Ratio	[95% Conf. Interval]	*P*-value
Age	1.07	1.06–1.09	< 0.001	1.06	1.05–1.07	< 0.001	1.08	1.07–1.09	< 0.001	1.06	1.05–1.08	< 0.001
Male	0.63	0.44–0.89	< 0.01	1.53	1.12–2.10	< 0.01	0.48	0.38–0.60	< 0.001	1.26	1.01–1.57	0.04
ISS	1.02	1.00–1.05	0.05				1.00	0.99–1.02	0.73			
NISS	1.02	1.00–1.03	0.02				1.00	0.99–1.02	0.39			
Polytrauma	0.46	0.26–0.83	< 0.01				0.57	0.40–0.82	< 0.01			
RTS	0.86	0.70–1.05	0.13				0.91	0.78–1.06	0.22			
Head and Neck AIS ≥3	3.04	1.95–4.73	< 0.001	1.79	1.13–2.84	0.01	2.00	1.54–2.60	< 0.001			
Thorax AIS ≥3	0.30	0.18–0.51	< 0.001				0.37	0.27–0.51	< 0.001			
Abdomen AIS ≥3	0.27	0.10–0.75	0.01				0.55	0.33–0.89	0.02			
Extremity AIS ≥3	0.68	0.41–1.14	0.09				0.94	0.69–1.28	0.69			
Low fall	10.96	6.37–18.86	< 0.001	3.48	2.06–5.87	< 0.001	7.60	5.63–10.26	< 0.001	2.35	1.43–3.88	< 0.01
Non-Low Fall	0.30	0.12–0.74	< 0.01				0.38	0.23–0.64	< 0.001			
Motor vehicle	0.11	0.01–0.76	0.03				0.12	0.04–0.38	< 0.001			
Motorcycle	0.03	0.00–0.18	< 0.001				0.06	0.03–0.13	< 0.001			
Pedestrian	0.19	0.03–1.41	0.11				0.39	0.15–0.97	0.04			
CCI 2^1^	4.96	3.08–8.01	< 0.001	2.26	1.38–3.70	< 0.01	3.93	2.66–5.78	< 0.001	1.77	1.14–2.74	0.01
CCI > =3^1^	7.85	4.36–14.15	< 0.001	3.36	1.98–5.69	< 0.001	7.66	4.53–12.95	< 0.001	3.53	2.39–5.22	< 0.001
Diabetes mellitus	1.91	1.28–2.85	< 0.01	1.31	1.68–2.52	0.04	2.05	1.55–2.71	< 0.001	1.30	1.05–1.60	0.02
Cancer	4.59	2.54–8.26	< 0.001	1.76	0.94–3.32	0.08	2.91	1.74–4.75	< 0.001			
Blood transfusion	0.24	0.08–1.77	0.02				0.35	0.19–0.66	< 0.01			
Orthopedic Operation	0.26	0.14–0.47	< 0.001				0.28	0.19–0.41	< 0.001			
Fluid and Electrolyte Disturbances	2.00	0.98–4.12	0.06				1.47	0.84–2.60	0.18			
Hospitalization > = 30 days	1.89	1.27–2.80	< 0.01	1.99	1.02–3.86	0.04	1.70	1.28–2.25	< 0.001	1.89	1.04–3.42	0.04

^1^Ref: Charlson Comorbidity Index of 0–1

**Fig. 1:**
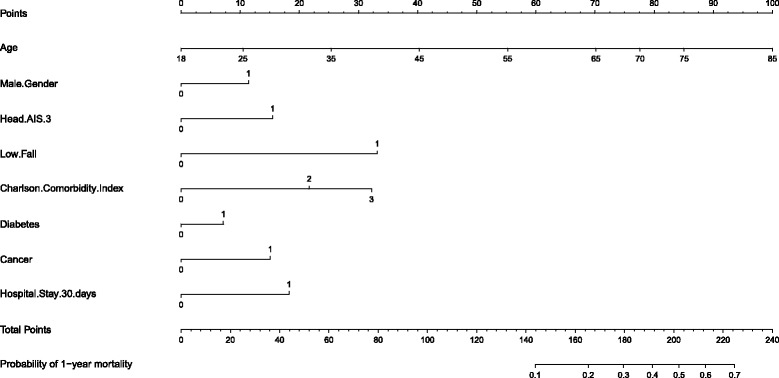
Nomogram Predictor for 1-year Mortality for Adult Survivors of Major Blunt Trauma

When the stepwise method for variable selection was applied for model selection, the multivariable model with the lowest Akaike information criteria showed: age (OR 1.06, 95% CI 1.05–1.07, *p* < 0.001), male gender (OR 1.53, 95% CI 1.12–2.10, *p* < 0.01), low fall from 0.5 m or less (OR 3.48, 95% CI 2.06–5.87, p < 0.001), Charlson comorbidity index of 2 (OR 2.26, 95% CI 1.38–3.70, p < 0.01), Charlson comorbidity index of 3 or more (OR 3.36, 95% CI 1.98–5.69, p < 0.001), diabetes (OR 1.31, 95% CI 1.68–2.52, *p* = 0.04), cancer (OR 1.76, 95% CI 0.94–3.32, *p* = 0.08), head and neck AIS 3 or more (OR 1.79, 95% CI 1.13–2.84, *p* = 0.01), length of hospitalization of 30 days or more (OR 1.99, 95% CI 1.02–3.86, p = 0.04) were predictors of 1-year mortality in the final model (Table 2). This model had a c-statistic of 0.85 and satisfied the Hosmer-Lemeshow goodness-of-fit test. We used the coefficients and independent variables in our model to generate the nomogram, whereby the linear predictor uses the coefficients and independent variables to calculate the risk of 1-year mortality (Fig. 1 nomogram).

**Fig. 2:**
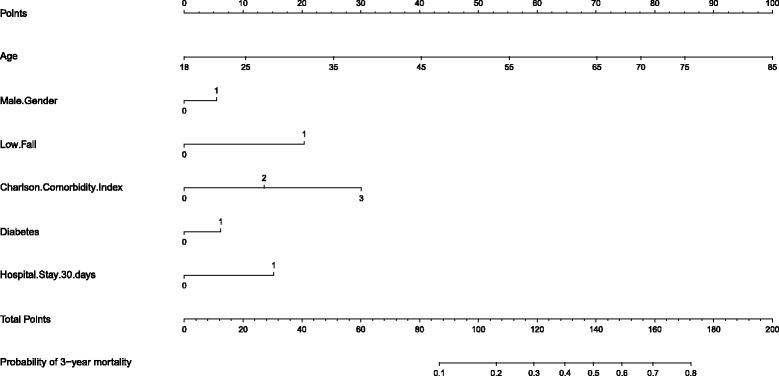
Nomogram Predictor for 3-year Mortality for Adult Survivors of Major Blunt Trauma

The analysis was repeated for 3-year mortality (Table 2). Similar factors were found significant for this model; however, head and neck AIS of 3 or more and cancer were no longer significant contributors to the model at 3 years, and inclusion of these factors in the multivariable model did not improve the c-statistic. This model had a c-statistic of 0.83 and satisfied the Hosmer-Lemeshow goodness-of-fit test, from which we generated the nomogram predictor for 3-year mortality (Fig. 2 nomogram).

**Fig. 3:**
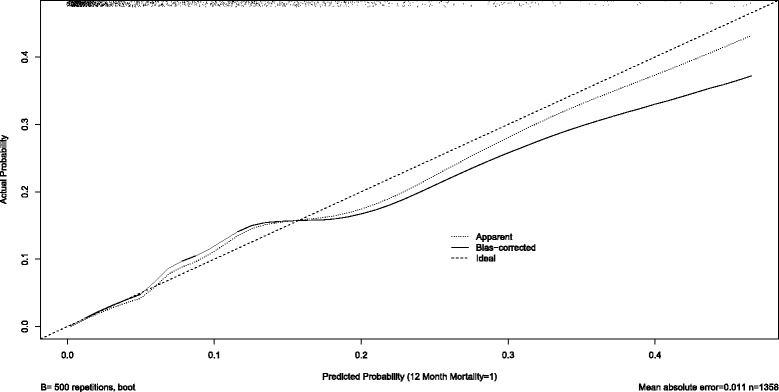
Calibration curves for 1-year Mortality for Adult Survivors of Major Blunt Trauma

**Fig. 4:**
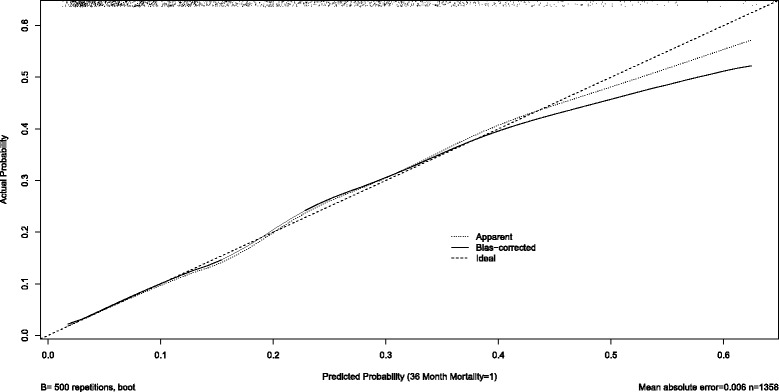
Calibration curves for 3-year Mortality for Adult Survivors of Major Blunt Trauma

Both models were validated on the second dataset, with an overall accuracy of 0.94 and 0.84 for 1-year and 3-year mortality respectively. When the 1-year mortality model was validated on the subgroup of patients aged under-55, the accuracy was 0.99, while for the subgroup of patients aged 55 and over, the accuracy was 0.88. For the 3-year mortality model, accuracy was 0.98 for the under-55, and 0.75 for the patients aged 55 and over. Figure 3 shows the calibration curve for 1-year mortality, showing adequate fit in the validation dataset, although boot-strapping (“bias-corrected” curve) suggests that the model was slightly over-confident in predicting death, especially for the lower-risk range of patients. Figure 4 shows the calibration curve for 3-year mortality, showing excellent model fit across the range of patients.

None of the sensitivity analyses changed our findings or improved model fit. In the 3-year mortality model excluding patients who were transferred in and whose admission was delayed for more than 24 h, the male gender odds ratio remained unchanged, but it was no longer statistically significant (*p* = 0.10).

### Observed vs expected mortality

The overall observed mortality of our study population was lower than the expected mortality based on the age- and sex-specific mortality rates for Singapore residents (Table 3). However, patients aged 55 and over had a higher than expected mortality. When the over-55 study population was grouped by major risk factors, into patients injured by low fall versus other blunt trauma, by head AIS score, or by Charlson comorbidity index, all the over-55 patients had a higher than expected mortality (Table 3).

**Table 3:** Observed vs Expected Mortality

			1-year survival probability	3-year survival probability
All adults	Overall	Expected	0.82	0.79
		Observed	0.93	0.84
	Non-Low Fall	Expected	0.83	0.80
		Observed	0.98	0.94
	Low Fall	Expected	0.77	0.72
		Observed	0.86	0.72
	Head and Neck AIS < 3	Expected	0.82	0.80
		Observed	0.96	0.90
	Head and Neck AIS > =3	Expected	0.81	0.78
		Observed	0.91	0.80
Aged 55 and over	Overall	Expected	0.93	0.85
		Observed	0.89	0.76
	Non-Low Fall	Expected	0.97	0.90
		Observed	0.96	0.88
	Low Fall	Expected	0.90	0.81
		Observed	0.85	0.70
	Head and Neck AIS < 3	Expected	0.94	0.88
		Observed	0.93	0.81
	Head and Neck AIS > =3	Expected	0.93	0.83
		Observed	0.87	0.74
	CCI 0–1	Expected	0.95	0.88
		Observed	0.91	0.80
	CCI > =2	Expected	0.83	0.70
		Observed	0.77	0.58

### Timing, location and cause of death

Of the deaths in the first year post-injury, only 10 of the 247 patients had injury mentioned in their death certificate. Nine listed trauma as the primary cause of death, almost all due to head injury. The majority of patients who died within the first year (216, 87.4%) had been injured in a low fall, which was the mechanism of injury for the majority of patients who died during the study period (Table 4). Many patients who died in the first year (200, 81.0%) had sustained an injury with a head and neck AIS score of 3 or more.

**Table 4:** Risk Factor Profile of Patients who died during Study period, by Time of Death

		Time of Death [Number (%) / mean (SD)]		
		Within 1 year	> 1 year, <=3 years	> 3 years post-injury
Age, mean (SD)		78.2 (12.6)	76.7 (14.3)	78.2 (11.0)
Male		140 (56.7)	159 (52.3)	100 (57.1)
Head and Neck AIS ≥ 3		200 (81.0)	222 (73.0)	126 (72.0)
Low Fall		216 (87.4)	227 (74.7)	137 (78.3)
Charlson Comorbidity Index	0–1	178 (72.1)	248 (81.6)	142 (81.1)
	2	44 (17.8)	34 (11.2)	19 (10.9)
	> = 3	25 (10.1)	22 (7.2)	14 (8.0)
Diabetes Mellitus		67 (27.1)	75 (24.7)	45 (25.7)
Cancer		25 (10.1)	17 (5.6)	9 (5.1)
Hospitalization > = 30 days		66 (26.7)	67 (22.0)	41 (23.4)

The top primary causes of death in the first year were pneumonia or respiratory (100, 40.5%), cancer (37, 15.0%), cerebrovascular (40, 16.2%) and cardiac (31, 12.6%). Most deaths (146, 59.1%) occurred in an acute hospital, with the remainder occurring in patients’ homes (73, 29.6%) or institutions (28, 11.3%). Of the patients dying in an acute hospital, pneumonia or respiratory causes were the commonest primary cause of death (68, 26.7%), followed by cardiac (24, 15.6%), cerebrovascular (14, 9.1%), cancer (14, 9.1%), and the remainder attributed to trauma, urinary tract infection, renal failure, gastrointestinal disorders, or other infections. The commonest primary causes of death for patients dying in an institution were cancer (11, 39.3%), and pneumonia or respiratory causes (10, 35.7%). Home deaths were mostly signed up as cerebrovascular (24, 32.9%), pneumonia or respiratory causes (25, 34.2%), and cancer (12, 16.4%).

Of the 304 patients who survived the first year post-injury but who had succumbed by 3 years, pneumonia or respiratory causes (126, 42.0%), cardiac (53, 17.7%) and cancer (46, 15.3%) were the top certified causes of death. The remaining primary causes of death (cerebrovascular, other infection, renal, gastrointestinal, etc.) each contributed less than 10% of the primary causes of death. Only seven of these deaths listed trauma in any of the primary or secondary causes of death. Similar proportions of deaths occurred in acute hospitals (188, 62.0%), patients’ homes (80, 26.7%), and institutions (31, 10.2%).

Of the 726 deaths occurring throughout the study period, when acute hospital deaths were compared to home or nursing home deaths, male patients were more likely to die in hospital (OR 1.48, 95% CI 1.10–1.84, *p* < 0.001), while older patients (OR 0.98, 95% CI 0.97–0.99, *p* < 0.001), head and neck AIS of 3 or more (OR 0.67, 95% CI 0.56–0.81, *p* < 0.001) and patients with cancer (OR 0.41, 95% CI 0.30–0,56, *p* < 0.001), were less likely to die in acute hospital.

## Discussion

Our study showed that age, gender, low fall injury mechanism (0.5 m or less), head injury, Charlson co-morbidity score, diabetes, cancer, and length of hospitalization predicted 1-year mortality for adult survivors of index hospitalization after blunt trauma of injury severity score of 12 or more. Many of these factors have been identified in studies of trauma survivors [1, 6], and some have also been shown to be risk factors for long-term survival for survivors of other critical illness [21]. Over 90% of our patients survived to 1 year, similar to other studies of survivors of severe injury [22, 23]. One long-term study of trauma survivors showed 90% living independently and 90% returning to work [24]. Our model had high predictive value for the 7% of patients who died within a year despite surviving the index hospitalization, with a c-statistic of 0.85, using only eight factors, most of which can be obtained from electronic medical records or discharge summaries. For 3-year mortality, most of the same factors remained significant, although head injury and cancer were no longer selected in the multivariable model. This suggests that the factors common to both models (age, Charlson index, diabetes, low fall), factors which are associated with frailty [25, 26], contributed to both one-year mortality as well as delayed mortality at 3 years, whereas head injury and cancer were major contributors for patients who succumbed earlier, within the first year. Interestingly, males were less likely to die on univariate analysis, but more likely to die in the multivariable model. This was probably because most young, high-velocity injuries were males, with an overall better prognosis, but once other factors like age, co-morbidities and mechanism of injury were included in the multivariable model, males had a worse prognosis than females.

Even though our study population was limited to patients sustaining major trauma (ISS of 12 or more [11]), the death certification did not reflect the burden of injury, potentially leading to an underestimation of the public health impact of injury [2].

We did not find overall injury severity to be predictive of long-term mortality, which was a risk factor in one large study with significant penetrating trauma patients [6], but not in another [9]. One reason is that we only focussed on patients with an ISS of 12 or more, shown in a recent study to be at increased risk of mortality [11]. Another difference is that we focussed on blunt trauma patients alone, whereas the other studies included significant numbers of penetrating trauma patients [6, 9]. Penetrating trauma patients are younger, are likely to be from higher-risk socioeconomic groups [27], and were at higher-risk of long-term adjusted mortality in these studies [3, 4, 6, 9]. This may also explain why, in our blunt trauma study population, we only found higher than expected mortality in the patients aged 55 and over, whereas studies with significant penetrating trauma showed higher than expected mortality for the entire study population [6].

Total length of hospital stay predicted long-term mortality in our study, as found in other studies [7, 9, 28]. It is likely that clinical factors (complications, slower recovery after injury or surgery), as well as social factors (known to affect the overall length of stay [29, 30]) both play a role in the predictive value of this variable in the model. Unfortunately, the only socio-demographic data available in our registry is race, which was not significant in our analysis.

Low falls are associated with poor long-term outcomes [31, 32], possibly due to physical frailty [10, 25, 26]. Frailty is increasingly recognised as an independent prognosticator in aging populations [33], and recurrent falls could contribute to delayed mortality [34]. In contrast, patients injured after a fall higher than 0.5 m did not have a worse outcome than other high-velocity blunt mechanisms of injury if they survived the index hospitalization, despite the overall high morbidity, poor functional outcome, and mortality associated with high falls [35, 36]. This is expected because patients strong enough to climb up to a higher height are likely to be functionally independent pre-injury, as might be expected of patients injured by other high-velocity mechanisms.

Diabetes mellitus was an independent predictor of 1-year mortality, in line with studies of severely injured patients [37, 38]. Head injured patients also had a worse prognosis [39, 40]. Discharge destination, a proxy for functional outcome [41, 42], was not predictive of 1-year mortality, contrary to the findings in some studies [6, 9]. Chronically ill patients may already have had support networks, which may explain why not all studies find discharge destination to correlate with function [42, 43]. In the Singapore context, home care may have been possible despite poor function if there was good caregiver support, with the widespread availability of domestic workers [44]. A more optimistic explanation is that patients transferred to a rehabilitation hospital could have obtained functional improvement and better eventual survival after rehabilitation [45, 46]. Due to the high proportion of missing data for functional outcome measures [47, 48] and quality of life [49] scores in the registry, we could not study these important factors.

The strengths of the study are the complete survival follow-up from the death registry for Singapore residents, and distinguishing high-risk low falls from lower-risk mid- or high-falls [10], made possible by the detailed fall height documentation in our registry. Unfortunately, we do not know whether this model applies to non-residents, a significant proportion of the Singapore population, as the death registry only captures information for residents, and non-residents may have been repatriated upon discharge from hospital for rehabilitation in their countries of domicile.

The main limitation, common to all registry-based studies, is the use of a database not designed to prospectively examine long-term outcomes. The model accuracy is higher for patients aged under 55 than for older patients. This could be explained by the many potential causes of death unrelated to trauma for the patients aged 55 and over, which may not have been diagnosed or present at the time of the index hospitalization for trauma. In addition, age, low falls and co-morbidities may not fully capture the extent of frailty or subsequent onset post-trauma. We plan to use this model to study high-risk patients in a future prospective study, and include measures of frailty, function, socioeconomic factors, educational level, social support and quality of life. High-risk patients may benefit from additional community-level support, which we hope to explore in a future study. Another limitation is that some groups of high-risk patients, e.g. young spinal injury patients, may be at higher risk of long-term mortality [50, 51], but manifesting in a time-frame longer than the 3 years captured by our study.

Singapore is an urban country in Asia. The relative proportions of blunt mechanism of injury (fall, road vehicle injury) appear similar to studies in other contexts [32, 47, 48]. Our model may be generalizable to settings with similar universal healthcare systems and aging populations dominated by blunt injury, and we hope that other registries will attempt validation of our model.

## Conclusions

Adult survivors of major blunt trauma can be risk-stratified at discharge for long-term support. Our predictive model is likely to be most relevant in urban aging populations.
